# Biochemical, Immunohistochemical, Histopathological, and Apoptotic Evaluation of Nickel Oxide Nanoparticle- and Microparticle-Induced Testicular Toxicity in Male Rats

**DOI:** 10.1021/acsomega.4c01005

**Published:** 2024-12-18

**Authors:** Caglar Adiguzel, Hatice Karaboduk

**Affiliations:** Faculty of Science, Department of Biology, Gazi University, Ankara 06500, Türkiye

## Abstract

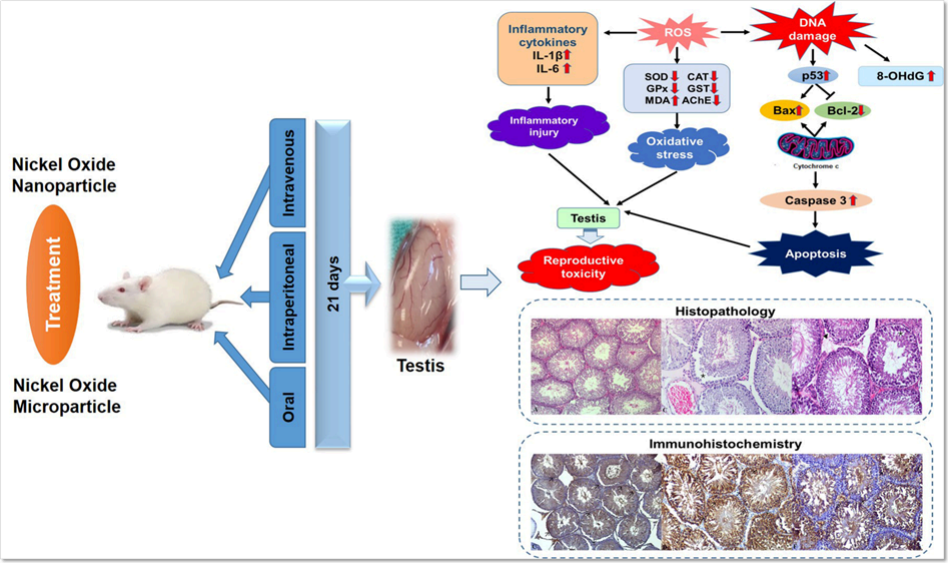

Nickel oxide nanoparticles are engineered particles that are now widely used in medicine, agriculture, and industry applications. This study aimed to investigate subchronic testicular toxicity induced by nickel oxide (NiO) and nickel oxide nanoparticles (NiONPs) in rats by comparing oral, intraperitoneal (IP), and intravenous (IV) routes of administration. Forty-two male Wistar rats were used for the study, and seven groups were formed: control group, NiO oral (150 mg/kg), NiO IP (20 mg/kg), NiO IV (1 mg/kg), NiONP oral (150 mg/kg), NiONP IP (20 mg/kg), and NiONP IV (1 mg/kg). At the end of the 21 day treatment, we collected the testicular tissue of rats to measure biomarkers such as oxidative stress, apoptotic, and inflammatory levels to observe histopathological and immunohistochemical changes. NiO and NiONP treatment caused a decrease in antioxidant activities and AChE levels, an increase in MDA, IL-1β, IL-6, and 8-OHdG levels, a decrease in Bcl-2 expression, and an increase in caspase-3, Bax, and p53 expressions in apoptotic markers. In addition to histopathologic changes in the testicular tissue, an increase in expression of the endoplasmic reticulum stress marker GRP78 was also observed. In conclusion, NiONPs (especially NiONP IV) increased testicular toxicity by disrupting the oxidant–antioxidant balance more than NiO microparticles.

## Research Highlights

1. Nickel oxide nanoparticles and microparticles caused testicular toxicity in male rats.
2. Oxidative stress plays an important role in nickel oxide-induced testicular toxicity.
3. Nickel oxide toxicity caused a decrease in antioxidant enzyme activities and an increase in apoptosis.
4. Testicular toxicity caused by nickel oxide nanoparticles was higher than microparticles.

## 1 Introduction

Nanotechnology is one of the most developing fields in recent years, and many nanomaterials have been produced and continue to be produced in the modern age.^1^ Nanoparticles are structures with different shapes and properties, with an average size of less than 100 nm.^2^ Thanks to the rapid developments in nanoengineering and technology, materials such as metal oxide nanoparticles and carbon nanotubes have been produced, and these structures have been used frequently in medicine, agriculture, and industry.^3^ Technological developments in the production of nanoparticles and subsequent widespread use in the industry have increased the exposure of these structures to water, soil, and nutrients.^4^ Although the assessment of nanoparticle toxicity is important since their accumulation in the environment will seriously affect human health, the degree of nanoparticle toxicity is directly related to these structures size, shape, and surface chemistry.^5^ Nanoparticle toxicity usually triggers oxidative stress, causing cellular molecules, DNA damage, and cell death in advanced injury.^6^ NiO nanoparticles consist of Ni^2+^ ions and O^2–^ ions arranged in crystal lattice structures and are frequently used in plastics, batteries, coatings, and blood glucose and urea level sensors in the medical field due to their magnetic properties and high conductivity.^7,8^ Like other nanoparticles, nickel oxide nanoparticles are a major concern for humans and animals due to their widespread use and environmental leakage. Exposure of nanoparticles to humans usually enters through the pulmonary, gastrointestinal, or cutaneous route, mixes with the blood, and disperses to organs from there.^9^ Because of their size, NiO nanoparticles are easily incorporated into the cell. Therefore, they cause more oxidative stress than microparticles.^10^ During absorption, they pass through the gastrointestinal tract, enter the circulation, and cause tissue injury and oxidative stress by affecting many systems, including hematological, genetic, renal, hepatic, and reproductive.^2,11^ It is known that chemical or environmental agents cause toxicity and damage to testicles and male gonads, which are the production sites of spermatogenesis and androgens. As a result, they cause deformations in the reproductive system.^12^ In a study investigating the effect of nickel nanoparticles on the reproductive system, according to the results of nickel application in male and female rats, it was found that uterine and ovarian tissues were injured in females, there were changes in the excretion of hormones such as follicle-stimulating hormone and estradiol, sperm motility decreased, and histopathological changes in seminifier tubules in male rats were observed to form.^13^ One of the main reasons nickel oxide is included in many industrial products is that it is a transition metal. Due to these properties, Ni ion toxicity arises from many nickel-containing products. However, it was stated that the level of nickel toxicity is related to the particle size, shape, and its solubility in water.^14^ The reason for cellular toxicities arising from nickel nanoparticles is the decrease in antioxidant activities due to the increase in reactive oxygen species (ROS). This situation creates oxidative stress with the increase in lipid peroxidation.^15,16^ Antioxidant enzymes play a very important role in clearing ROS in the cellular mechanism.^17,18^ Oxidative stress, which is increased in the deficiency of antioxidants or the cell defense mechanism, is associated with the progression of diseases such as those in the reproductive system and cancer.^19^ Studies have shown that oxidative stress disrupts the cell cycle, damages structures such as proteins and lipids, causes oxidative DNA damage, increases inflammation, and triggers cell death.^20−22^ In experimental studies, oral gavage applications are frequently used in rats and mice, and it is one of the methods with clear results.^23^ There are many successful studies in which the oral gavage method was applied to experimental animals.^18,24^ One of the routes of administration widely used in rodent studies is the intraperitoneal method. In this way of administration, solutions can be sent to the animals quickly and in large volumes, and the stress level of the animals is minimal.^25^ Intravenous administration, on the other hand, is the method that provides high-volume delivery of substances without being attached to the first pass barrier in the application to animals.^26^ In the literature search, the number of studies on the reproductive toxicity of NiONP and NiO is not very large. So far, there are several available studies on the reproductive toxicity of nickel oxide nanoparticles. In addition, Noshy et al. (2022) studied the protective role of hesperidin against testicular toxicity caused by nickel oxide nanoparticles.^2^ Considering these studies, there is still a need for complementary information on the mechanism of toxicity of NiONP and NiO in the male reproductive system of rats. Therefore, the current study aims to compare both the application routes and the toxicity caused by nano- and microsized nickel oxide nanoparticles and microparticles in the reproductive system of male rats.

## 2 Materials and Methods

### 2.1 Reagents

Nickel oxide nanoparticles (NiONPs) (CAS number: 1313-99-1) and nickel oxide (NiO) microparticles (CAS number: 1313-99-1) were procured from Nanogarific Nano Technology (METU/Teknokent, Ankara, Turkey).

### 2.2 Characterization of Nickel Oxide Microparticles

Our previous study carried out the characterization of nickel oxide nanoparticles.^27^ X-ray diffractometry (XRD) analyses of NiOMPs were performed on a Bruker D8 Advance device with CuKα (λ = 1.5418 Å) beam at a scanning speed of 0.03° per second in the range of 20–90°. The surface morphology of NiOMPs was determined using a JEOL JSM 6060 LV scanning electron microscope (SEM).

### 2.3 Animals and Treatment Schedule

The study was carried out with the approval of the Gazi University Animal Experiments Local Ethics Committee (protocol no: G.U. ET-21.033). In the study, 42 male Wistar rats with a body weight of 250–300 g were used and these rats were obtained from the Gazi University Laboratory Animal Breeding and Experimental Research Center. Rats were kept in special cages, fed a standard laboratory diet, and given water with six rats in each cage. Stock solutions were created to apply nickel oxide (NiO) microparticles and nickel oxide nanoparticles (NiONPs). These stock solutions were prepared in physiological saline and sonicated with an ultrasonicator for 30 s before each application of NiONPs. Selected doses of NiO microparticles and NiONPs were administered to experimental animals orally,^28^ intraperitoneally,^15^ and intravenously.^26^ The 42 male Wistar rats used in the experiment were divided into seven groups (Table 1).

**Table 1:** Design of Experimentals

groups	
Group I	control group (1 mg/kg bw distiled water)
Group II	NiO oral application group (150 mg/kg bw per day)
Group III	NiO intraperitoneal administration group (20 mg/kg bw per day)
Group IV	NiO intravenous administration group (1 mg/kg bw per day)
Group V	NiONP oral application group (150 mg/kg bw per day)
Group VI	NiONP intraperitoneal administration group (20 mg/kg bw per day)
Group VII	NiONP intravenous administration group (1 mg/kg bw per day)

At the end of 21 days of experimental applications, the testicular tissue of rats was quickly removed with the combination of ketamine and xylazine. For histological studies, testicular tissues were stored in 10% formaldehyde. For oxidative stress parameters, testicular tissues were washed in sodium phosphate buffer and quickly placed in a −80 °C. Testicular tissues were stored at −80 °C until they were used for molecular studies in QIAzol.

### 2.4 Measurement of Organ Weights

At the end of the 21 day experimental period, the testes of the rats were removed. The testes of the control and treatment groups were measured with an automatic weight measuring device (ANDGX-600, Japan). The right testis of the experimental animals was used for weighing.

### 2.5 Acetylcholinesterase (AChE) Activity Analysis of Testicular Tissue

AChE activity of testicular tissue was quantified spectrophotometrically at 412 nm by acetylthiocholine iodide as a substrate following the procedure of Ellman et al. (1961).^29^ Activity was assayed by increasing the amount of 5-thio-2-nitrobenzoate, a yellow anion formed by reacting thiocholine with 5,5-dithiobis(2-nitrobenzoic acid) (DTNB).

### 2.6 Estimation of Oxidative Stress Markers

For oxidative stress analysis, the testes of rats were primarily homogenized in phosphate-buffered saline (PBS) and centrifuged at +4 °C (12,000 rpm, 15 min). The supernatants obtained were used for the determination of the enzyme activities. These analyses were performed by purchasing enzyme-linked immunosorbent analysis (ELISA) commercial kits from the BT LAB (Bioassay) Technology Laboratory. The kit coded (Cat. No: E0156Ra) was used to determine the malondialdehyde (MDA) level by following the manufacturer’s instructions. To determine antioxidant enzyme activities, superoxide dismutase (SOD) (Cat. No: E1444Ra), catalase (CAT) (Cat. No: E0869Ra), and glutathione peroxidase (GSH-PX) (Cat. No: E1172Ra) and glutathione S-transferase (GST) kits with the code (Cat. No: E0513Ra) were used. MDA levels and enzyme activities were measured as ng/mL at 450 nm using an ELISA reader.

### 2.7 Analysis of Interleukin 1 Beta (IL-1β) and Interleukin-6 (IL-6) Levels in Testicular Tissue

To determine the levels of IL-1β and IL-6 in the testicular tissue of rats, we used rat interleukin-1 beta (Cat. No: E0119Ra) and rat interleukin-6 (Cat. No: E0135Ra) ELISA kits from BT LAB were used after the collected tissues were homogenized with phosphate buffer. To obtain the supernatant, the homogenates are centrifuged at 12,000 rpm for 15 min at 4 °C. IL-1β and IL-6 levels were determined following the manufacturer’s instructions. Finally, IL-1β and IL-6 levels were measured at 450 nm by a microplate reader.

### 2.8 Analysis of Oxidative DNA Damage in Testicular Tissue

The level of 8-hydroxy-2′-deoxyguanosine (8-OHdG) in testicular tissue was determined using an Elabscience (Cat No: E-EL-0028) kit. Following the manufacturer’s kit instructions, rat testicles were homogenized in phosphate buffer, necessary conjugates, substrate solutions, and stop solutions were added, and their concentration at a wavelength of 450 nm was measured.

### 2.9 mRNA Expression Analysis by qRT-PCR

mRNA expression of Bax (Bcl-2 associated X protein), Bcl-2 (B cell lymphoma-2), Cas-3 (Caspase 3), and p53 was determined using real-time PCR. Total RNA isolation from tissues was performed using a QIAzol Lysis Reagent (Qiagen, Germany), and quantification of RNA samples was verified by a NanoDrop 2000 (Thermo Fisher Scientific). cDNA was generated from mRNA using the RT2 First Strand Kit (Qiagen, Germany). RT^2^ SYBR Green ROX FAST Mastermix and Bax, Bcl-2, Cas-3, p53, and Actin-beta (Actb) (housekeeping gene) (Qiagen, Germany) primers were used to measure qRT-PCR reactions on a Rotor-Gene Q (QIAGEN, Germany). To avoid errors due to manipulation, all samples were duplicated. The data were normalized using the values obtained from the control groups' average, and *Ct* values were calculated with the “delta delta *Ct*” (ΔΔ*Ct*) method.

### 2.10 Histology

For histopathological examination, the testicular tissues were dissected, and the tissue samples were fixed in 10% neutral formalin solution for 24 h, processed by using a graded ethanol series, and embedded in paraffin. The paraffin sections were cut into 5–6 μm-thick slices and stained with hematoxylin and eosin for light microscopy examination. Ten slides were prepared from each testicular tissue. Each testicular tissue preparation was examined under a microscope, and the status of histopathological changes was evaluated. Photographs of the prepared sections were taken with a Leica DFC295 camera attached to a Leica DM 100 light microscope.

### 2.11 Immunohistochemical Assay

All sections were mounted on adhesive (poly-l-lysine) slides to measure GRP78 immunoreactivity in testicular tissue. The sections were dehydrated and deparaffinized by being passed through the xylol and alcohol series. To prevent antigen masking in the sections, antigen recovery was achieved by placing the sections in citrate solution in a microwave oven for 5 min. After washing the sections three times in phosphate buffer (PBS) for 5 min each, we soaked them in hydrogen peroxide (H_2_O_2_) for 15 min to inhibit the endogenous peroxide phase. Ultra V Block was added to the sections with 5 min waiting time. In the next step, GRP78 (Elabscience, Cat No: E-AB-60037) antibody was diluted at 1:100 and added to the tissues. After the antibody was added to the sections, the sections were kept at +4 °C overnight. Then, secondary antibody was added to the sections, which were kept in PBS 3 times, and 3 × 5 with PBS was applied again. The immune reaction was then amplified using a streptavidin–avidin–peroxidase complex and rehydrated in PBS. DAB solution was added to the sections, and the sections were allowed to absorb chromogen well for 15 min. Gill hematoxylin was used for counterstaining, and then, sections were washed in water, passed through an alcohol series and xylol, and covered with Entellan. The preparations were studied under a light microscope (Leica DM 100), and immunoreactivity was measured using the ImageJ program (ImageJ 1.53k).

### 2.12 Statistical Analysis

SPSS program version 22 and GraphPad prism version 8 were used to compare the data obtained from the molecular and biochemical results. Study data were evaluated by ANOVA and Tukey tests. Statistically, *p <* 0.05 values were considered significant.

## 3 Results

### 3.1 Characterization of NiOMPs

**Figure 1:**
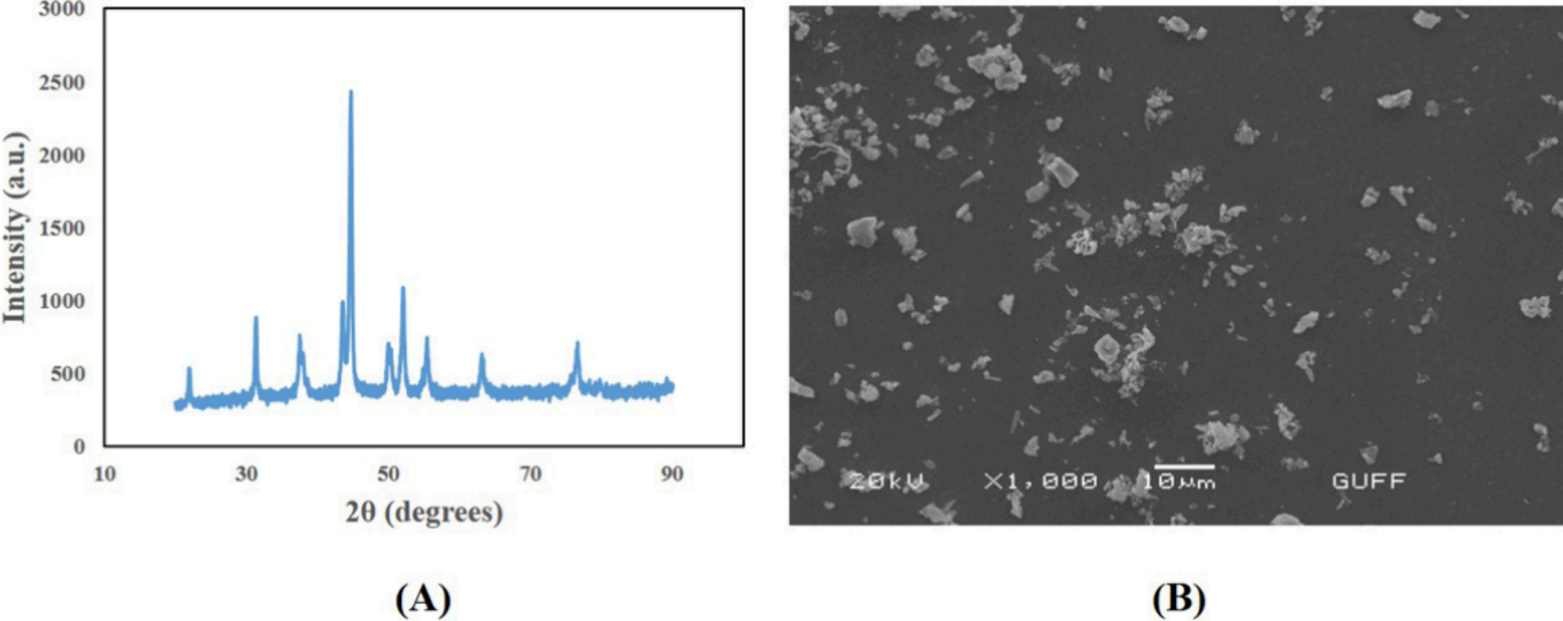
(A) XRD diffractogram of NiOMPs and (B) SEM image of NiOMPs.

The intensity of the peaks in the analysis of NiO microparticles by XRD is consistent with the intensity of JCPDS card no: 01-080-5508. As a result of the study, diffraction peaks 22, 31, 50, 51, and 55° were obtained in the XRD graph shown in Figure 1A. SEM examination of NiO microparticles showed that they have rough, cubic, and spherical shapes (Figure 1B).

### 3.2 Evaluation of Organ Weights

During the 21 day experiment, no death was observed in any experimental group. Testicular weights were measured, and no significant difference was observed between the control and treatment groups (*p* > 0.05) (Table 2).

**Table 2:** Relative Testis Weights of Control and Experimental Rats^a^

parameters	control	NiO oral	NiO IP	NiO IV	NiONP oral	NiONP IP	NiONP IV
relative testis weight (g/100 g body weight)	1.24 ± 0.09	1.15 ± 0.10	1.23 ± 0.21	1.12 ± 0.11	1.07 ± 0.07	1.16 ± 0.07	1.11 ± 0.08

a Values are means ± SD for six rats in each group. Significance at *p* > 0.05.

### 3.3 Results of the Acetylcholinesterase Activity and MDA Level

**Figure 2:**
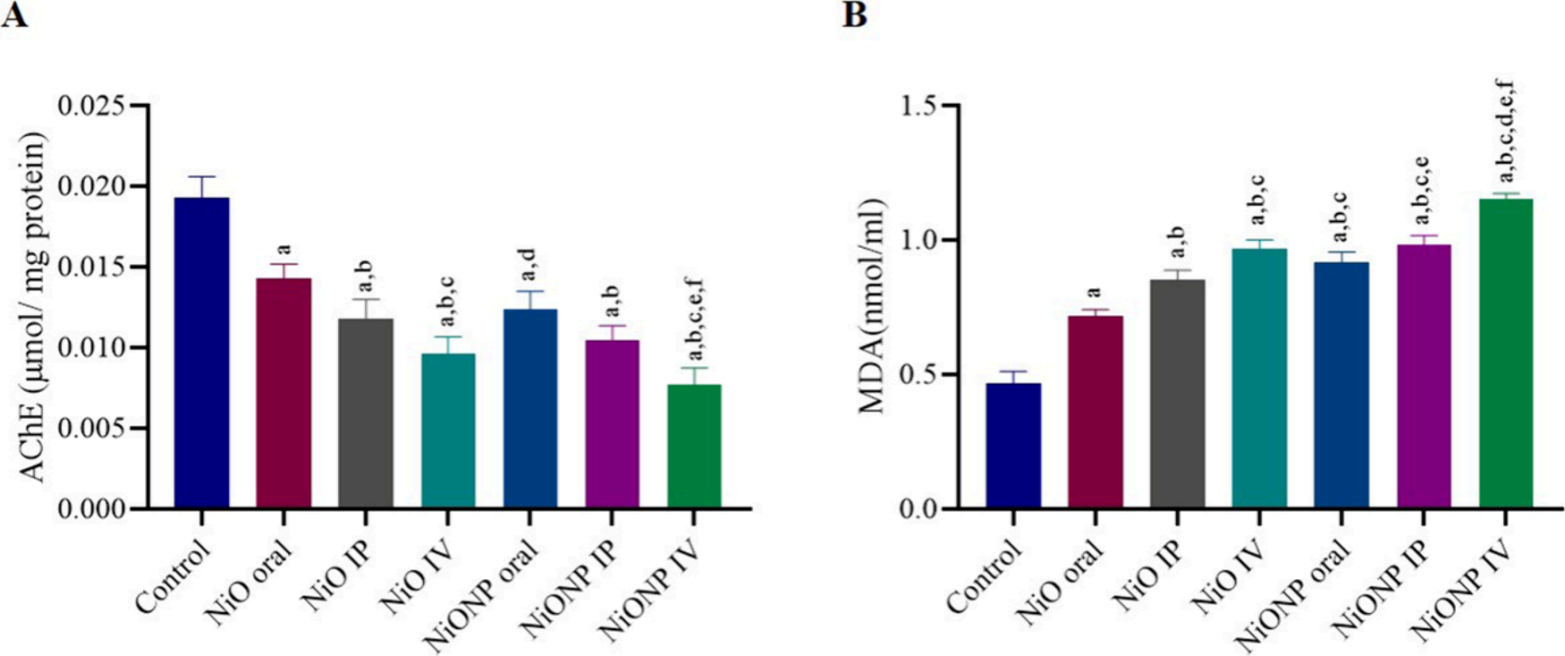
(A) Acetylcholinesterase activity and (B) MDA (nmol/mL) levels in testis. ^a^Significant difference between the control group and other groups. ^b^Significant difference between the NiO oral group and other groups. ^c^Significant difference between the NiO IP group and other groups. ^d^Significant difference between NiO IV and other groups. ^e^Significant difference between NiONP oral and other groups. ^f^Significant difference between NiONP IP and other groups. (*n* = 6). Significance at *p* < 0.05. The difference between the level of significance was determined by the Tukey test using one-way ANOVA.

The activities of AChE in the testis tissue of all groups are recorded in (*p* < 0.05) (Figure 2A). Testis AChE activities significantly declined in the NiO microparticle and NiO nanoparticle groups when contrasted to the control group. Among the micro- and nanoparticle-treated groups, the highest effect was observed in the groups in which IV administration was performed. The highest decrease was found in the NiONP IV group.

NiO microparticle and nanoparticle toxicity was associated with augmented lipid peroxidation, evidenced by a significant elevation in the MDA level. When the groups to which nickel oxide microparticles were applied were evaluated among themselves, the group with the highest increase in the MDA level was NiO IV, while when the groups to which nickel oxide nanoparticles were applied were compared, the highest increase was in the NiONP IV group. The findings of this study show that NiONP IV administration causes the most effective increase in the MDA level compared to administrations in other groups (*p* < 0.05) (Figure 2B).

### 3.4 Results of Oxidative Stress Markers

**Figure 3:**
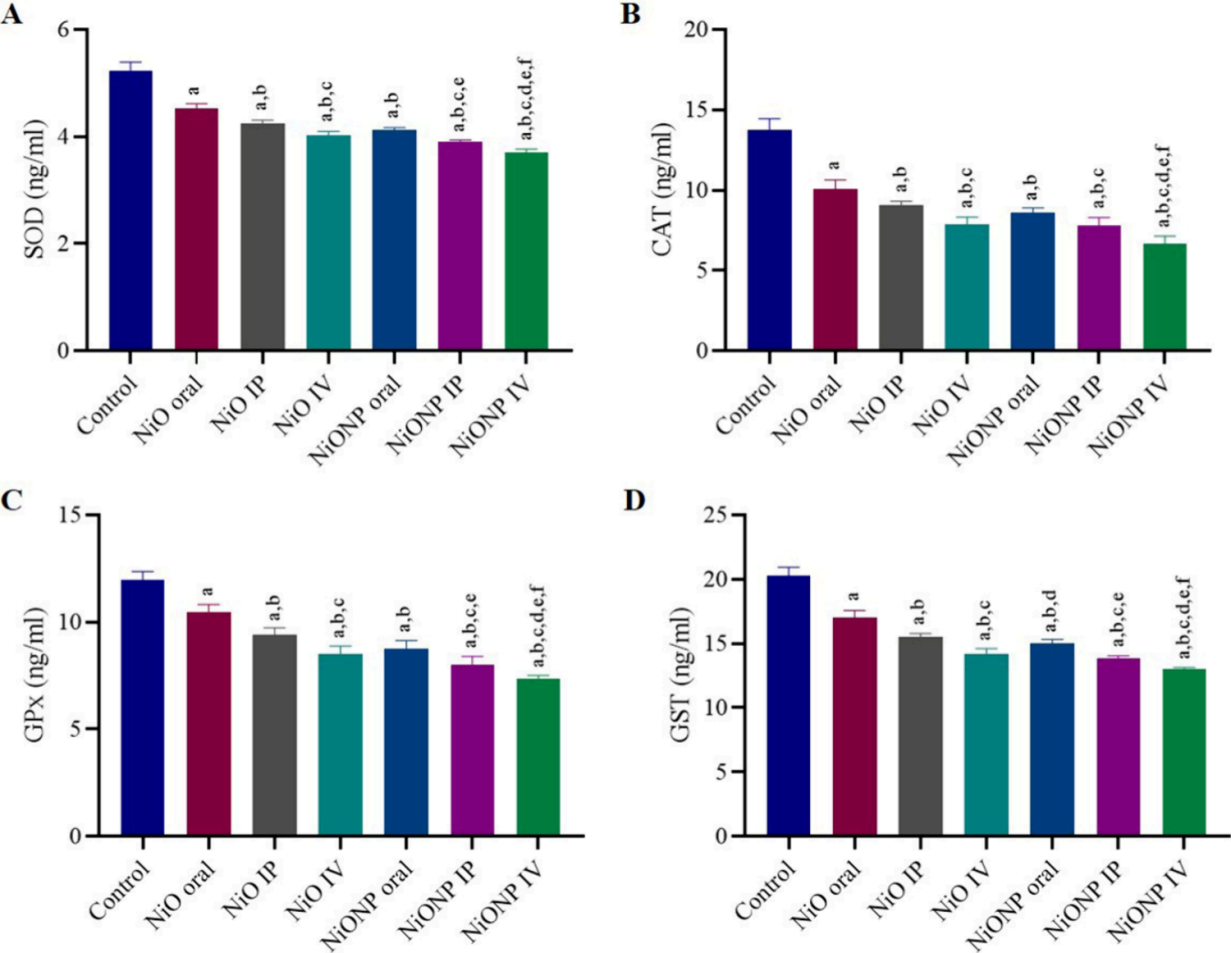
(A) SOD activity in the testis. (B) CAT activity in testis. (C) GPx activity in testis. (D) GST activity in testis. ^a^Significant difference between the control group and other groups. ^b^Significant difference between the NiO oral group and other groups. ^c^Significant difference between the NiO IP group and other groups. ^d^Significant difference between NiO IV and other groups. ^e^Significant difference between NiONP oral and other groups. ^f^Significant difference between NiONP IP and other groups. (*n* = 6). Significance at *p* < 0.05. The difference between the level of significance was determined by the Tukey test using one-way ANOVA.

As a result of the evaluation made to determine the redox profile in testicular tissue, it showed a decrease in antioxidant enzyme activities after NiO microparticle and NiO nanoparticle application compared with the control group. When the antioxidant activities of SOD, CAT, GPx, and GST were examined, NiO IV was the group in which the antioxidant activities decreased the most among the NiO microparticle groups. NiONP IV is the group where the antioxidant activities decrease the most among the NiO nanoparticle groups. The highest decrease among all groups was NiONP IV (*p* < 0.05) (Figure 3A–D).

### 3.5 Analysis of Oxidative DNA Damage

**Figure 4:**
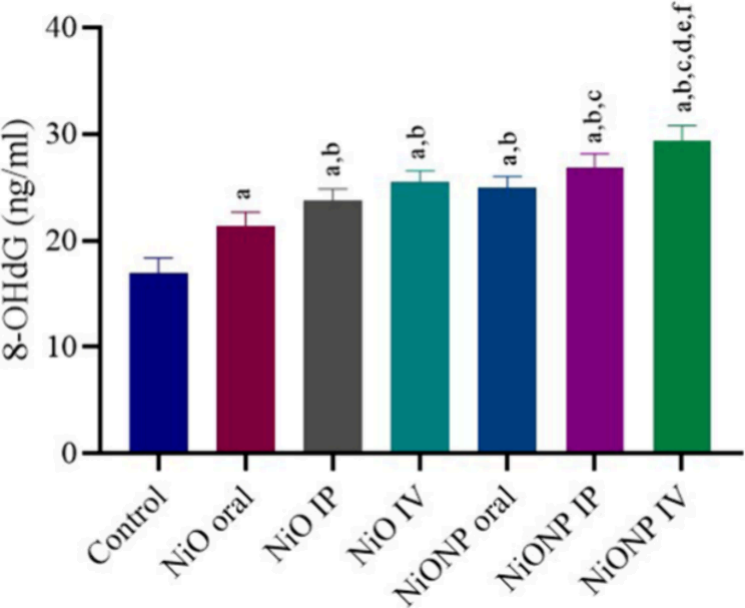
8-OHdG level in testicular tissue. ^a^Significant difference between the control group and other groups. ^b^Significant difference between the NiO oral group and other groups. ^c^Significant difference between the NiO IP group and other groups. ^d^Significant difference between NiO IV and other groups. ^e^Significant difference between NiONP oral and other groups. ^f^Significant difference between NiONP IP and other groups. (*n* = 6). Significance at *p* < 0.05. The difference between the level of significance was determined by the Tukey test using one-way ANOVA.

According to the data obtained in the study, the lowest 8-OHdG level was in the control group. Statistical difference was observed in NiO- and NiONP-treated groups compared to the control group (*p* < 0.05). Among the nickel oxide microparticle- and nickel oxide nanoparticle-treated groups, NiONP IV was the group that caused oxidative DNA damage in the testicular tissue and increased the 8-OHdG level the most (*p* < 0.05) (Figure 4).

### 3.6 Analysis of IL-1β and IL-6 Activity

**Figure 5:**
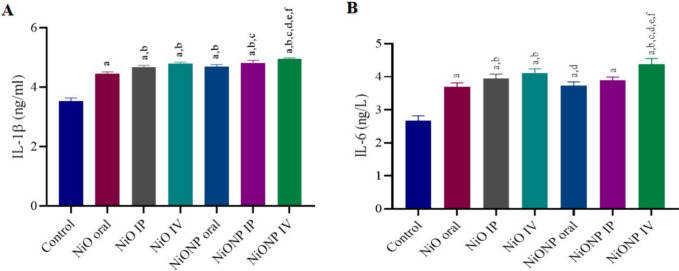
IL-1β (A) and IL-6 (B) levels in testicular tissue. ^a^Significant difference between control group and other groups. ^b^Significant difference between NiO oral group and other group. ^c^Significant difference between NiO IP group and other groups. ^d^Significant difference between NiO IV and other groups. ^e^Significant difference between NiONP oral and other groups. ^f^Significant difference between NiONP IP and other groups. (*n* = 6). Significance at *p* < 0.05. The difference between the level of significance was determined by the Tukey test using one-way ANOVA.

At the end of the experimental applications, the IL-1β activity in rat testicles was evaluated. A significant increase was observed in the IL-1β level in the groups treated with nickel oxide microparticles and nickel oxide nanoparticles compared to the control group (*p* < 0.05). When the groups to which nickel oxide microparticles were applied were evaluated among themselves, it was determined that the NiO IP and NiO IV groups were statistically different from the NiO oral group (*p* < 0.05). No significant difference was observed between NiO IP and NiO IV groups. It was observed that there was a significant difference in NiONP IP and NiONP IV in the nickel oxide nanoparticle-treated groups compared to NiONP oral (*p* < 0.05). When the NiONP IV group was compared to the NiONP IP group, the inflammation was higher and significantly different in the NiONP IV group. NiONP IV was observed statistically in the group with the highest IL-1β level among all groups (*p* < 0.05) (Figure 5A).

When the IL-6 level in the rat testicles was examined, a statistically significant increase was observed in the activity in the NiO- and NiONP-applied groups compared to the control group (*p* < 0.05). While a significant difference was observed in the NiO IP and NiO IV groups between the NiO-administered groups compared to the NiO oral group, no statistical significance was observed between these two groups. When the NiONP groups were compared within themselves, statistical significance was found in the NiONP IV group compared to the NiONP IP and NiONP IV groups. Among all groups, the group with the highest IL-6 level was statistically the NiONP IV group (*p* < 0.05) (Figure 5B)

### 3.7 Analysis of Apoptotic Markers

**Figure 6:**
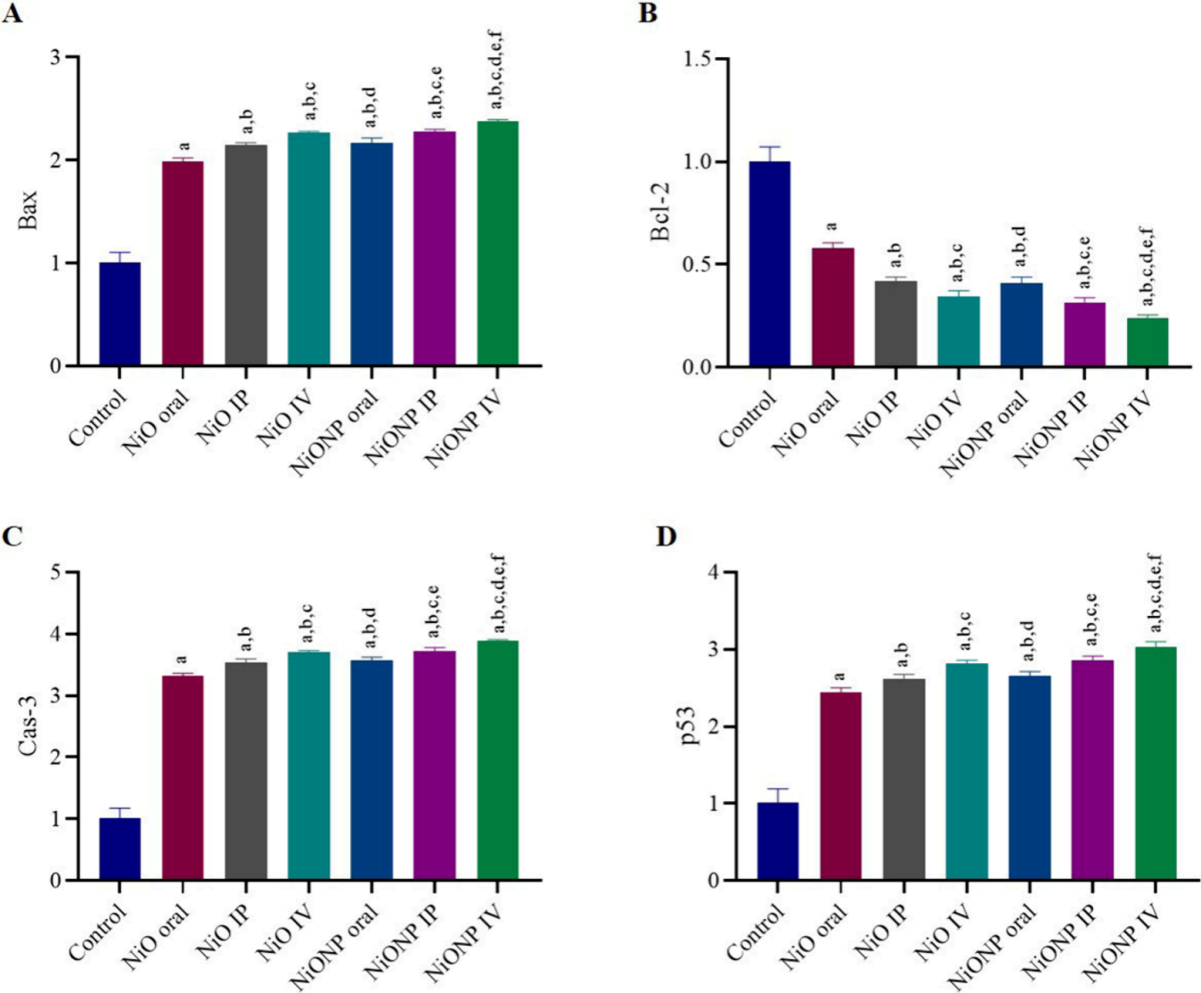
(A) Bax status in the testis. (B) Bcl-2 status in testis. (C) Cas-3 status in testis. (D) p53 status in testis. ^a^Significant difference between the control group and other groups. ^b^Significant difference between the NiO oral group and other groups. ^c^Significant difference between the NiO IP group and other groups. ^d^Significant difference between NiO IV and other groups. ^e^Significant difference between NiONP oral and other groups. ^f^Significant difference between NiONP IP and other groups. (*n* = 6). Significance at *p* < 0.05. The difference between the level of significance was determined by the Tukey test using one-way ANOVA.

After 21 days of nickel oxide and nickel oxide nanoparticle application, a significant upward increase was observed in caspase-3, Bax, and p53 activities in testicular tissue compared to the control group, while a significant decrease was found in Bcl-2 activity compared to the control group (*p* < 0.05) (Figure 6A–D). When the groups to which the nickel oxide microparticles were applied were evaluated among themselves, the increase in Bax, Cas-3, and p53 activity was the highest in the NiO IV group, while the decrease in Bcl-2 activity was the greatest in the NiO IV group. The NiONP IV group is the group with the highest increase in Bax, Cas-3, and p53 expressions in nickel oxide nanoparticle-applied groups. The group with the largest decrease in Bcl-2 expression was also the NiONP IV group. When the NiO microparticle- and NiONP-applied groups were evaluated among themselves in terms of apoptotic markers, it was observed that the group with the highest upstream Bax, Cas-3, and p53 expressions was NiONP IV. At the same time, the group with the highest decrease in Bcl-2 expression was also NiONP IV (*p* < 0.05) (Figure 6A–D).

### 3.8 Histological Changes in Testes

**Figure 7:**
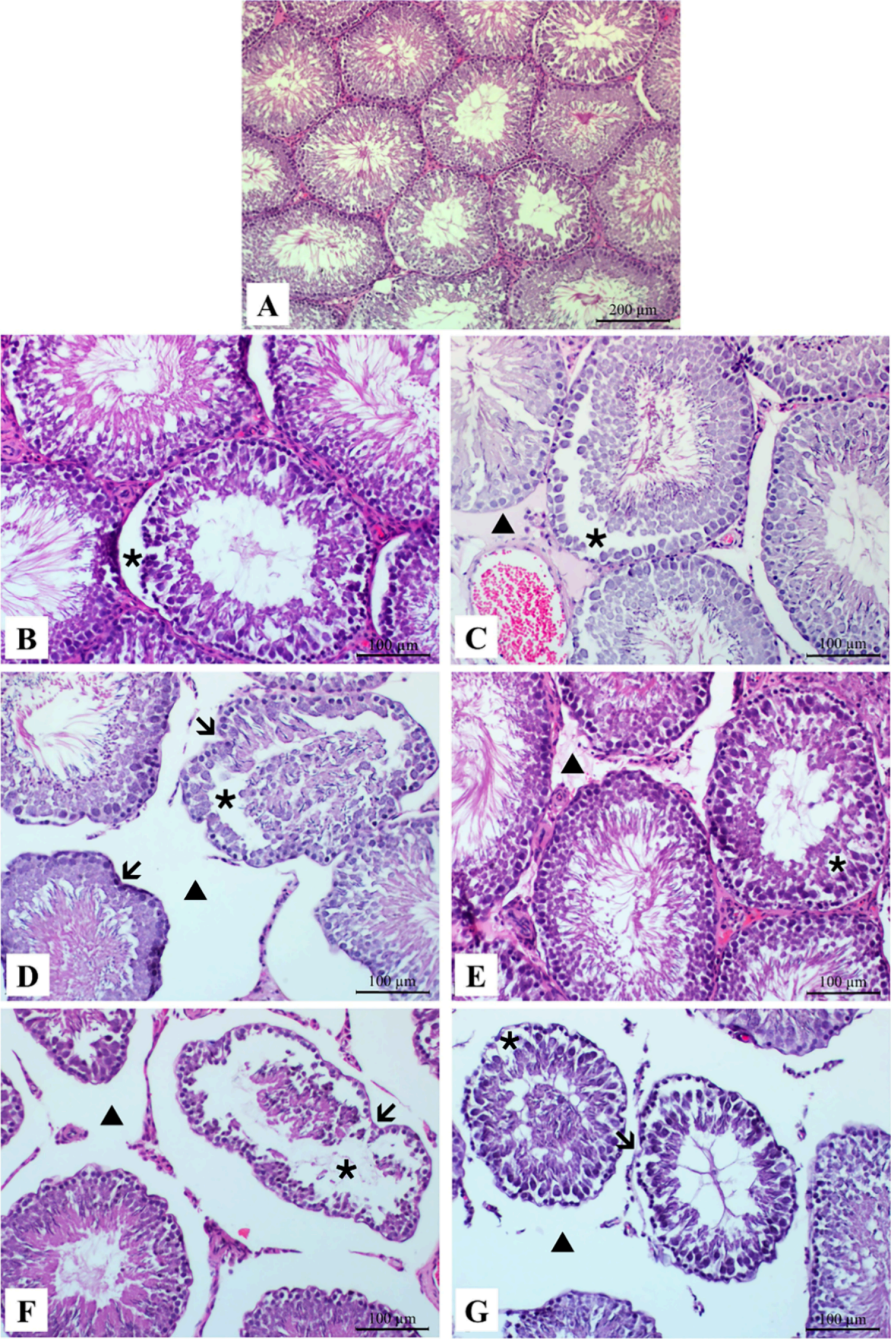
Histopathology of testicular tissues of NiO- and NiONP-treated rats. H&E. (A) Control group: no pathology observed, normal histologic structure. (B) Degeneration (star) in seminiferous tubules in the NiO oral exposure group. (C) Degeneration (star) in seminiferous tubules and edema (triangle) in interstitial area in the NiO IP exposure group. (D) Degeneration (star), irregular indentations (arrows) in the seminiferous tubules, and edema (triangle) in the interstitial area in the NiO IV exposure group. (E) Degeneration (star) in seminiferous tubules and edema (triangle) in the interstitial area in the NiONP oral exposure group. (F) Degeneration (star), irregular indentations (arrow) in the seminiferous tubules, and edema (triangle) in the interstitial area in the NiONP IP exposure group. (G) Degeneration (star), irregular indentations (arrow) in the seminiferous tubules, and edema (triangle) in the interstitial area in the NiONP IV exposure group.

Histopathologic changes in testicular tissue are listed in Figure 7. The seminiferous tubules and spermatogenic cells of the testicular tissue in the control group appeared normal (Figure 7A). Degeneration was observed in the seminiferous tubules in the testes of all groups treated with NiONP and NiO microparticles (Figure 7B–G). However, edema occurred in the interstitial space in the testicular tissues of NiOIP, NiOIV, NiONP oral, NiONP IP, and NiONP IV groups (Figure 7C–G, respectively). Irregular indentations were observed in the seminiferous tubules in the testes of NiO IV-, NiONP IP-, and NiONP IV-treated groups (Figure 7D, F, and G, respectively).

### 3.9 Immunohistochemical Findings of GPR78 for Testicular Tissue

**Figure 8:**
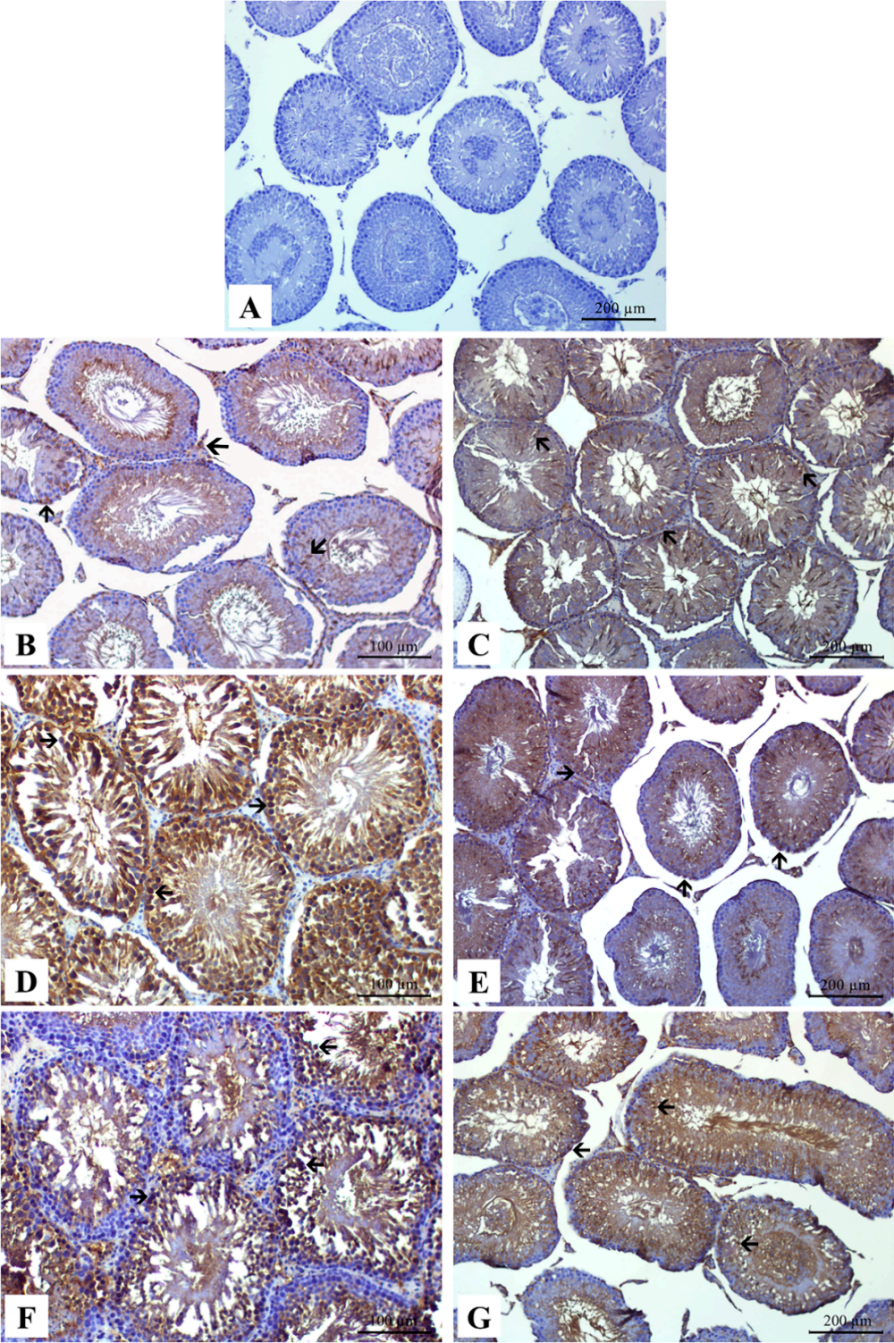
Immunohistochemical staining of GRP78 in the experimental groups. The seminiferous tubules of (B) NiO oral, (C) NiO IP, (D) NiO IV, (E) NiONP oral, (F) NiONP IP and (G) NiONP IV groups showed a strong increase in GRP78 expression (A) compared to the seminiferous tubules in the control group. Black arrows indicate immunoreactive cells.

**Figure 9:**
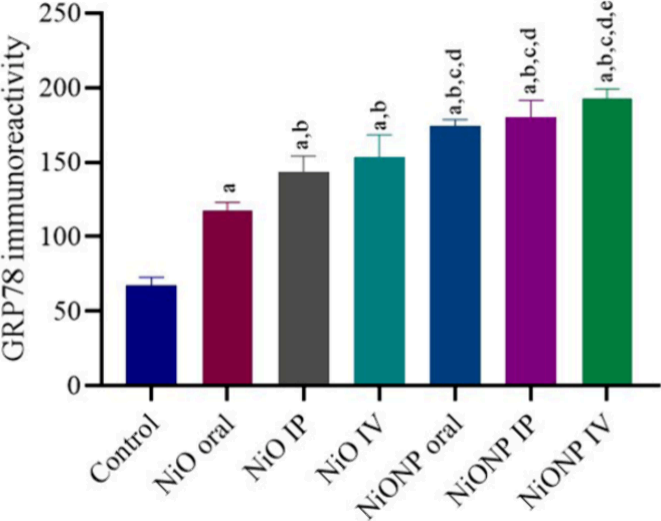
Statistical analysis of GRP78 expression in testicular tissue of the experimental groups. ^a^Significant difference between the control group and other groups. ^b^Significant difference between the NiO oral group and other groups. ^c^Significant difference between the NiO IP group and other groups. ^d^Significant difference between NiO IV and other groups. ^e^Significant difference between NiONP oral and other groups. (*n* = 6). Significance at *p* < 0.05. The difference between the level of significance was determined by the Tukey test using one-way ANOVA. *p* < 0.05.

We evaluated GRP78 expression in testicular tissue in immunohistochemical analysis (Figure 8). GRP78 expression was not detected in the control group testicular tissues (Figure 8A). In immunohistochemical examination, a significant increase in GRP78 expression in the germinal cells of the seminiferous tubules of the NiO- and NiONP-applied groups was observed compared to the control group (Figure 8B–G). A significant statistical difference was observed in the NiO IV and NiO IP groups compared to the NiO oral group (Figure 8B–D). A significant increase in GRP78 expression was observed in the groups where nickel oxide nanoparticles were applied, compared to the groups where nickel oxide microparticles were applied (Figure 8E–G). Figure 9 shows the statistical analysis of GRP78.

## 4 Discussion

The effects of heavy metals on the environment and living health have reached serious levels. When drinking water is contaminated with heavy metals, it is stated that arsenic, cadmium, nickel, mercury, chromium, zinc, and lead have become an important health problem for the environment and living things.^30^ Heavy metals can disturb the body’s metabolic functions through various ways. It causes oxidative stress in cells, triggering various diseases. Moreover, they may accumulate in vital body organs such as the liver, heart, kidney, and brain disturbing normal biological functioning.^30^ Some heavy metals such as cadmium and arsenic are known to be endocrine disruptors.^31^ It is also stated that heavy metals have negative effects on insulin-stimulating hormone and carbohydrate metabolism.^32^ Nickel and its derivatives used in this study are also among the heavy metals.^27^

Nickel oxide nanoparticles are nanoengineered structures produced in various shapes and sizes and frequently used in different industrial products.^33^ XRD analysis in our previous study showed that the NiONP is well crystallized. SEM examination of NiONPs indicated that the shape structures were spherical and round.^27^ In this study, it was observed that the peaks obtained in the XRD analysis of NiO microparticles were different from those of NiONPs. In our SEM examinations, NiO microparticles were observed to be of various shapes.

The public health and environmental problem caused by its excessive use is a cause for concern, and examining the toxicological, toxicokinetic, dose–time relationship, and other potential hazards of this metal in many ways can only be addressed through experiments on a mammalian species.^34^ It has been shown that with their unique chemical and physical properties, the possible damage and toxicities of nickel-derived nanoparticles in biological structures differ from microparticles from the same elemental composition.^19,35^ Studies have shown that nanoparticles can pass through living membranes and the blood-testicular barrier.^36^ It has been stated that nanoparticles entering the circulation can be displaced in organs such as the liver, kidney, testis, and spleen.^37^ It has been observed that these nanoparticles entering the body cause toxicity by accumulating in the testicles, epididymis, or reproductive organs over time.^38^ Body weight and organ weight data are known to be important in toxicological studies.^13^ In our previous study, it was reported that there was no significant change in rat weights.^27^ In the current study, relative testicular weights were also evaluated, and no significant difference was observed between the control and treated groups.

The neurotransmitter acetylcholine, which plays an important role in nervous system function, is hydrolyzed by AChE. AChE could be a target for toxins, leading to the inhibition of its activity. Changes in AChE activity are considered as an important marker in determining the neurotoxicity of various pollutants.^39^ The findings of this study show that NiO microparticles and nanoparticles inhibit AChE activity in the testes and that this inhibition is most pronounced in the NiONP IV group. Although not many studies suggest that the inactivation of AChE enzymes is due to the occupation of their active sites by nickel oxide, some studies confirm the observations of our study.^28,40^

When the oxidant and antioxidant balance in living things is disturbed, oxidative stress occurs, which seems important in metal toxicity.^41^ It is known that metallic nanoparticles also cause a free radical increase, leading to lipid peroxidation in the cell and altering the activity of antioxidant enzyme systems.^15,42^ The increase in lipid peroxidation caused by oxidative stress causes tissue injuries, and the indicator of the increase in lipid peroxidation in the cell is determined by the increase in MDA concentration.^17^ In the current study, a significant increase in the MDA level was observed in the groups treated with NiO microparticles and NiONPs, compared to the control group. The highest increase occurred in the NiONP IV group. We can say that this increase in the MDA level is due to the damage caused by reactive oxygen species in cell membranes. The results of other studies are consistent with the results of our study.^15,29^ To prevent oxidative stress, mammalian cells have enzymatic and nonenzymatic antioxidant systems interacting with ROS and preventing and neutralizing its increase.^43^ It is known that nanoparticles cause excessive ROS production, leading to deterioration in mitochondrial functions, changes in antioxidant activities, cytotoxicity, and tissue injuries.^3,44^ SOD plays a role in protecting against the damaging effects of superoxide radicals and is effective in the conversion of superoxide radicals to hydrogen peroxide.^45,46^ CAT, together with SOD, forms the first line of defense and plays a role in the conversion of hydrogen peroxide to water and oxygen.^2^ On the other hand, GPx and GST act as detoxifiers by converting xenobiotics such as hydrogen peroxide into nontoxic structures.^47^ In the current study, SOD, CAT, GPx, and GST antioxidant activities in testicular tissues of rats in the groups to which NiO microparticles and NiO nanoparticles were applied decreased compared to those of the control group. The greatest decrease in activity among all groups occurred in the NiONP IV group. The reductions in these antioxidant activities can be attributed to differences in the routes of administration. In addition, the activities of SOD, CAT, GPx, and GST may have decreased because they take part in detoxifying radicals such as superoxide, hydrogen peroxide, and hydroxyl to protect cells and tissues from oxidative damage.^48,49^ The results of our study show compatibility with other studies done in the past.^19,50^ Among the DNA bases, guanine is the most sensitive to oxidative damage caused by free radicals. 8-OHdG is the most widely used biomarker to show ROS-mediated DNA damage.^51,52^ In the current study, there was a significant increase in the 8-OHdG level of the NiO microparticle- and NiO nanoparticle-applied groups in the control group. The highest increase was observed in the NiONP IV group. This increase in DNA damage can be attributed to the increase in reactive oxygen species caused by the NiO microparticles and NiONPs. Studies show that nickel oxide nanoparticles cause DNA damage in liver and kidney tissues in rats.^53^ In another study, it was observed that an increase in the 8-OHdG level occurred as a result of oxidative damage in kidney tissue in rats to which nickel oxide microparticles and nanoparticles were administered, which was in line with our results.^3^ Cytokines are 20–30 kDa polypeptide or glycoproteins produced and secreted by stimulated monocytes, lymphocytes, macrophages, and various other cells and are involved in regulating immune response and inflammation.^54^ Inflammation is a biological response to repair damaged or damaged tissues where reactive oxygen species are excessively increased and antioxidant defense is inhibited in the body. In this process, inflammatory cytokines [such as interleukin 1 beta (IL-1 β) and interleukin 6 (IL-6)] are released to regulate inflammatory responses.^55,56^ IL-1β is a cytokine that plays an important role in the pathogenesis of inflammatory diseases and damages that occur under oxidative stress and also plays a key role in initiating inflammation.^57^ The current study showed a significant increase in IL-1β levels in the NiO microparticle- and NiONP-applied groups compared to the control group. The group with the highest IL-1β level was the NiONP IV group. The increase in the IL-1β level can be interpreted as the increase in the inflammatory cell flow of the body against the oxidative stress caused by the NiO microparticle and NiO nanoparticle application in the testicular tissue. Although IL-1β level studies related to different application routes of NiO microparticles and NiO nanoparticles in testicular tissue are not very common in the literature, an increase in IL-1β levels was observed in NiONP-applied studies, and this is consistent with our results.^58,59^ IL-6 is among the proinflammatory cytokines, such as Tnf α and IL-1β. Like other proinflammatory cytokines, IL-6 has been reported to mediate the movement and activation of cells to the area of inflammation, the development of various diseases, and the spread of inflammation.^60,61^ In this study, IL-6 levels increased statistically in the NiO- and NiONP-applied groups compared with the control group. The group with the highest IL-6 level among the administration groups is the NiONP IV group. Oxidative damage caused by nickel oxide microparticles and nanoparticles in the testicular tissue of rats increased the IL-6 level and IL-1β. The results of changes in IL-6 levels related to toxicities caused by nickel oxide are in parallel with our studies.^58,60,61^

Studies have shown that NiONP application causes oxidative stress by causing an increase in ROS in cells and causes apoptosis in the cell by causing damage to the DNA helix.^21^ Apoptosis is programmed cell suicide involving changes in the cytoplasm, nucleus, and cell membrane associated with different biochemical processes.^11^ In the cell undergoing apoptosis, vacuole formation in the cytoplasm, swelling in the endoplasmic reticulum, and chromatin condensation in the nucleus are observed, and the cell loses its communication with its surroundings.^11,62^ In ROS-induced oxidative stress, mitochondria damage and dysfunction are significant factors in the pathway leading to cell death.^63,64^ It has been stated that intracellular ROS accumulations damage essential organelles, such as mitochondria and endoplasmic reticulum, and disrupt macromolecules. Furthermore, as a result, it has been stated that apoptosis is initiated in the cell. The apoptotic process generally proceeds via the extrinsic or intrinsic pathways (mitochondrial pathways).^65^ Although apoptosis is considered the main mechanism of nanoparticle (NP)-induced cell death, the intrinsic mitochondrial apoptotic pathway plays an important role in metal oxide NP-induced cell death.^33^ p53 tumor suppressor protein is the determinant of cell fate. p53 regulates the expression of genes involved in cell cycle regulation, DNA replication and repair, and maintenance of homeostasis.^66^ When the cell is damaged or under oxidative stress, if the repair mechanisms fail to normalize the cell, p53 leads the cell to apoptosis.^67^ While Bcl-2 is an antiapoptotic protein localized in the inner membrane of the mitochondria, nucleus, and endoplasmic reticulum, Bax is usually localized in the cytoplasm and functions as a pro-apoptotic protein by initiating the apoptotic cascade in the cell under oxidative stress.^68,69^ Although the Bax/Bcl-2 ratio determines apoptosis, as this ratio increases, cytochrome c release from mitochondria increases and caspase-9 is activated. Caspase-9 also activates caspase-3, causing cell death.^70^ In the current study, we evaluated the oxidative damage caused by NiO microparticle and NiO nanoparticle application in testicular tissue with biomarkers Bax, Bcl-2, caspase-3, and p53. When we look at the results of the current study, it was seen that the most affected group was NiONP IV. This may be due to the effect of the route of administration of NiONP. The literature search found no study on testicular tissue, including the comparative toxicity of oral, intraperitoneal, and intravenous NiO microparticle and NiO nanoparticle administration. The results of other studies showing that NiO microparticles and NiO nanoparticles cause oxidative stress in testicular tissue and lead cells to apoptosis show parallelism with our results.^19,29^

In toxicological studies, assessing the condition of tissues and organs is crucial. In histopathologic studies, nickel oxide accumulates in tissues and causes changes in the histological structure. Depending on the exposure and dose, nickel oxide particles can be distributed to different organs through the circulatory system.^40^ NiO nanoparticles have been reported to cause disturbances in the function and regulation of the cell structure in the testes of rats and to cause shedding and reduction of the epithelium in the seminiferous tubules. This shows that NiONPs can cross the blood-testis barrier.^2,71^ In the rat testis, administration of NiONPs induces rupture of the seminiferous tubules and disruption of the basement membranes.^37^ In another study, both nickel nano- and microparticles caused disorganization in the seminiferous tubules of rat testes and induced apoptosis in the cells.^13^ In the present study, NiO and NiONPs caused degeneration in seminiferous tubules and edema in the interstitial area. Histopathologic changes in tissues may vary depending on the amount of substance administered, time, electrical charge, and size.^2,34^ In this study, pathologic findings were observed in the testicular tissues of all groups treated with NiO nano- and microparticles.

For eukaryotic cells, the endoplasmic reticulum is an organelle that plays a vital role in protein folding and modification and contains enzymes and chaperones necessary for protein synthesis.^72^ When the function of this organelle is impaired under a series of unfavorable conditions, misfolding or unfolding of proteins occurs, and endoplasmic reticulum stress occurs.^73^ Studies have shown that oxidative stress, apoptosis, and endoplasmic stress are interconnected and that there is a relationship between endoplasmic reticulum stress and testicular toxicity.^73−75^ In the current study, we observed the expression of GRP78, an endoplasmic reticulum stress marker, in testicular tissue. By immunohistochemical staining, we observed that GRP78 levels in testicular tissue increased in NiO- and NiONP-treated groups compared to those in the control group. The statistically significant increase in NiONP groups compared with NiO groups was evidence that NiONPs caused more toxicity. Previous studies have rarely studied GRP78 expression levels in testicular tissue with NiONP administration.

In light of the data obtained in the present study, it was observed that NiONPs caused more damage to testicular tissue than NiO. Considering the routes of administration, it was determined that the intravenous route caused more toxicity in the testicular tissue. NiO and NiONPs were found to decrease antioxidant enzyme activities, increase lipid peroxidation, cause inflammation, and change apoptosis markers in testicular tissue.

In this study, the subacute testicular toxicity of NiO and NiONPs was investigated. Depending on the dose and route of administration, NiONPs and NiO disrupted the antioxidant–oxidant balance, induced oxidative stress, induced apoptosis, and caused histopathologic changes. To fully understand the effect of NiONPs and NiO on the reproductive system, chronic studies, studies on the effect on the female reproductive system, and studies in pregnant rats are needed to understand the effect on the offspring. We believe that our study can guide further studies. However, based on the results of this study, precautions should be taken against occupational and environmental effects of NiO and NiONPs.

## Data Availability

All data on the conclusion of testicular toxicity in this study are presented in the manuscript.
